# National Health Insurance Notes and Comments

**Published:** 1922-12

**Authors:** 


### NATIONAL HEALTH INSURANCE NOTES AND COMMENTS.

**Work as a Tonic.**—In the Government Actuary's Report on the Valuation of Approved Societies appears the following illuminating commentary on the fall in the claims for sickness benefit payments during the war:—

> Employment was abundant during the war years and wages were high. With such conditions operating universally and exceeding in marked degree the corresponding attributes of a state of industrial activity due to any normal cause, a decline in sickness claims was almost inevitable. The actual decline proceeded further, however, than even the prevailing economic conditions might reasonably be held to explain, and the fact that concurrently with the decline improved administrative methods were being applied in many quarters, frequently under pressure exercised by the Insurance Commissioners, does not suffice to bridge the difference. The beneficial factor which is explained by neither economic conditions nor efficient administration is the universal "will to work" which, under the stress of national necessity, dominated the civilian population during the years in which the fate of the country hung in the balance. The temperamental element, which plays so large a part in controlling physical capacity for work, came fully into operation, and in the right direction. Moreover, the post-war sickness experience of approved societies seems to show that this universal effort was not accompanied by any untoward reactions; on the contrary, the claims have not, since 1918, reached the pre-war level. That the resolution to work, based upon the trust reposed in its defenders, which carried the civilian community through the long years of war was beneficial, on the whole, from the point of view of health, is strongly supported by the experience of the approved societies, whose membership covers the great majority of the wage-earning population.

**Medical Benefit Developments.**—In a most interesting paper by Mr. Alexander Clark, the Clerk to the Aberdeen County Insurance Committee, read at the Conference of the Scottish Associations of Insurance Committees at Montrose, there were brought into sharp contrast the views of Mr. Frederick Hoffman, of U.S.A., and those of many authorities in London, Sussex, Nottingham, Glasgow, and elsewhere who were competent to speak with authority, and who spoke highly of the work of the panel doctors and of their relations with Insurance Committees. Mr. Hoffman, whose diligent research in every corner of these isles is well remembered by many busy people, was able to find all that he was looking for, and, fortified by the letters of growsers in the columns of the medical press, and by the medical correspondent of the "Times," went home to America, and wrote that "the unfortunate consequences of National Health Insurance may continue to blight the social and economic welfare of the British people for generations to come."

Mr. Clark examines the question of the development of the medical service for the insured, with particular reference to the proposals of the Consultative Council set up by the Scottish Board of Health. Auxiliary services are clearly essential to the improvement of the present scheme of medical benefit. But there is little hope of these auxiliary services being "provided at the expense of the State." And we doubt whether Mr. Clark is on safe ground in supposing that, with the auxiliary services administered by the local Health Authority, medical benefit would continue as a separate piece of administration in the hands of the Insurance Committees. In a scheme of extended services provided mainly at the expense of those who benefit by them, there are possibilities, and when such a scheme materialises, we agree that the "existing machinery for the administration of medical benefit should be grafted into the new system." But the effective trend must be towards unification of all health services under one local authority.

**Nursing for Insured Persons.**—In our November issue a letter appeared from Mr. West, the Clerk to the Cheshire Insurance Committee, stating that a scheme drawn up by that Committee for providing a nursing service for insured persons had had to be abandoned for lack of support from the Approved Societies. From the columns of the "National Insurance Gazette," we gather that the matter has been the subject of an animated correspondence between Mr. West and Mr. Rockliff, the tone of which hardly seems likely to assist in promoting a better understanding in the matter between Approved Societies and Insurance Committees.

It is clear from the letter from Mr. Gill Hodgson, the Clerk to the Liverpool Insurance Committee, which appears in this issue, that in Liverpool also very little support is at present forthcoming from the Societies. But Mr. Hodgson very sensibly takes the view that it is no good waiting for a comprehensive scheme for the whole country "which is not within the realm of practical politics." "That," Mr. Hodgson says, "is no reason why attempts should not be made to solve the problem on however small a scale, in order that the advantage of experience may be derived." This we believe to be sound advice. It is difficult to understand why Approved Societies should not make use of the local organisation which already exists for putting into force a scheme, however modest. The Societies are not, for the most part, geographically grouped, and they have no organisation in any area ready to administer any scheme for the benefit generally of insured persons in the area. The machinery of the Insurance Committee is in existence; the Societies, if they provided the bulk of the funds for nursing would clearly have a controlling voice on any *ad hoc* Committee formed to administer the nursing service. The doctors, through their Panel Committee, would see—as the Liverpool scheme provides—that undue demands were not made by individual doctors on the nursing service available, and the nursing arrangements would be linked up with the general scheme of medical benefit. It is difficult to understand why so essential a service as that of providing nursing assistance in the homes of the insured should have proved "not at all popular," as the "National Insurance Gazette" reported some little time ago when dealing with the question of the disposal of the Societies' surpluses disclosed on the valuation. It is no sufficient answer to say that a Society cannot contribute to a nursing scheme in one area which is not available in every area, because clearly the insured members to whom a nursing scheme was not available, could receive an equivalent benefit.

**Insurance Committees and a Wider Outlook.**—Health developments may seem remote when the general cry is for peace and tranquillity. But such developments would not, of necessity, involve State expenditure, and Insurance Committees and their staffs will do well to widen their outlook and maintain an interest in the general field of public health. The address given by Dr. Chalmers, the Medical Officer of Health for the City of Glasgow, at the Annual Conference of the Scottish Association of Insurance Committees, on the problem of venereal disease, and the paper read by Mr. Crew, the Clerk to the Leicestershire Insurance Committee, on "Health Propaganda," at the Conference of Clerks to Insurance Committees, are both indications that the Associations have some appreciation of the need for this wider outlook. The Leicestershire Insurance Committee in their annual report, recently published, refer to the exceptionally useful work that they have been able to do in arranging illustrated health lectures for school children with the co-operation of the Education Authorities and doctors, and the help of cinema proprietors.

**The Doctors and the Approved Societies.**—We do not think that Dr. Ridley Bailey need attach too much importance to the fact that the remarks of the retiring President of the National Association of Insurance Committees, Sir Alfred Warren, M.P., were received with applause, when he said that panel patients got much worse treatment now than under the old Friendly Society arrangements. Dr. Bailey, speaking at Coventry, would have the doctors arm to the teeth because of this hostility. But the Insurance Committees at any rate are surely not hostile. It is merely that criticisms of any class of persons always get roundly applauded at a public meeting. If the occasion had been a presentation to a distinguished member of the medical profession and it had been said that every medical man was an angel in disguise, there would probably have been hearty British cheers. And in any case, it may have been noted that the incoming President, Sir Wm. Glyn Jones paid a tribute to the work of the doctors, and said that the insured public received treatment such as it was impossible for them to get in the days before the Insurance Act. Such conflicting statements are a little bewildering, but they seem to average out pretty well.

